# An online training and feedback module enhances the musculoskeletal examination performance of medical interns

**DOI:** 10.1186/s12909-024-05683-w

**Published:** 2024-08-23

**Authors:** Matías Arteaga, Catalina Vidal, Cristián Ruz, Raúl Zilleruelo, Ernesto Pino, Javier Dauvergne, Pablo Besa, Sebastián Irarrázaval

**Affiliations:** Orthopedics Department, School of Medicine, Pontificia Universidad Católica de Chile, Diagonal Paraguay #362, Santiago, 8330077 Chile

**Keywords:** Medical education, Physical examination, Undergraduate, Distance learning, Musculoskeletal diseases

## Abstract

**Background:**

Pathologies of the locomotor system are frequent and can cause disability and impact the quality of life of the people affected. In recent years, online training and feedback have emerged as learning tools in many fields of medicine.

**Objective:**

This study aims to evaluate medical interns’ musculoskeletal examination performance after completing an online training and feedback module.

**Methods:**

This study employed a quasi-experimental design. Medical interns were invited to complete a 4-week musculoskeletal physical examination training and feedback module via an e-learning platform. The course included written and audiovisual content pertaining to medical history, physical examination, and specific tests for the diagnosis of the most common knee, spine, shoulder, ankle, and foot conditions. Before and after completing the module, their ability to perform the physical examination was evaluated using an objective structured clinical examination (OSCE) with simulated patients that took place face-to-face. A control group of experts was assessed using the OSCE, and their performance was compared to that of the interns before and after the training. At the end of the module feedback on the OSCE was provided to participants through the platform asynchronously and two evaluation questions about the user experience were conducted at the end of the study.

**Results:**

A total of 35 subjects were assessed using the OSCE, including 29 interns and 6 experts. At the beginning of the training module, the group of interns obtained an average score of 50.6 ± 15.1. At the end of the module, 18 interns retook the OSCE, and their performance increased significantly to an average of 76.6 ± 12.8 (*p* < 0.01). Prior to the training, the experts performed significantly better than the interns (71.2 vs. 50.6; *p* = 0.01). After the interns received the training and feedback, there were no significant differences between the two groups (71.2 vs. 76.6; *p* = 0.43). Two evaluation questions were conducted at the end of the study, revealing that 93% of the participants affirm that the training module will be useful in their clinical practice, and 100% of the participants would recommend the training module to a colleague.

**Conclusion:**

The online training and feedback module enhances the musculoskeletal examination performance of medical interns.

**Supplementary Information:**

The online version contains supplementary material available at 10.1186/s12909-024-05683-w.

## Background

Pathologies of the locomotor system are frequent and can cause disability and impact the quality of life of the people affected. In the United States, 50% of people over 18 years old and 75% of people 65 years of age and over suffer from a musculoskeletal disorder [1]. In addition, as many as 25–50% of visits to general practitioners are related to musculoskeletal syndromes, spine and knee pain are among the most common causes of health care visits [2, 3].

Physical examination is key with regard to musculoskeletal conditions [4]. Every general practitioner should perform a thorough physical examination to diagnose frequent and urgent musculoskeletal disorders when providing initial medical care [5, 6]. However, current evidence suggests that general practitioners do not perform adequate physical examinations, and they report feeling insecure when diagnosing or treating patients with these disorders [7, 8]. Accordingly, a high proportion of conditions that could be diagnosed and managed by general physicians are instead referred to orthopedic specialists. Furthermore, some studies have shown that as many as 42% of trauma referrals are unnecessary leading to increased wait times, system costs, and reduced efficiency [9, 10].

In recent years, online training and feedback have emerged as learning tools in many fields of medical education. In fact, undergraduate students and residents report achieving similar or superior results with respect to the acquisition of clinical skills from online training than from traditional methods as well as higher satisfaction rates [11, 12]. As students’ curricular time is limited and increasing clinical requirements reduce the time available for in-person learning of patient care [13, 14], it is essential to rethink traditional teaching methods. New approaches should focus on learning that combines diverse activities and includes effective feedback that can also adapt to the limited time of teachers. With this goal in mind, the present study aims to evaluate medical interns’ musculoskeletal examination performance after they complete an online training and feedback module.

## Methods

This study was conducted in 2021 and featured a quasi-experimental design. Medical interns from a single university program were invited to participate. Interns are final-year medical students who have completed all their theoretical courses but have not yet done their undergraduate clinical practice. The inclusion criteria were that the participants were required to be regular last-year medical students and to voluntarily sign an informed consent form prior to the start of the study. The exclusion criteria were having taken additional courses or elective training in musculoskeletal examination that were not included in the standard medical curriculum.

**Fig. 1:**
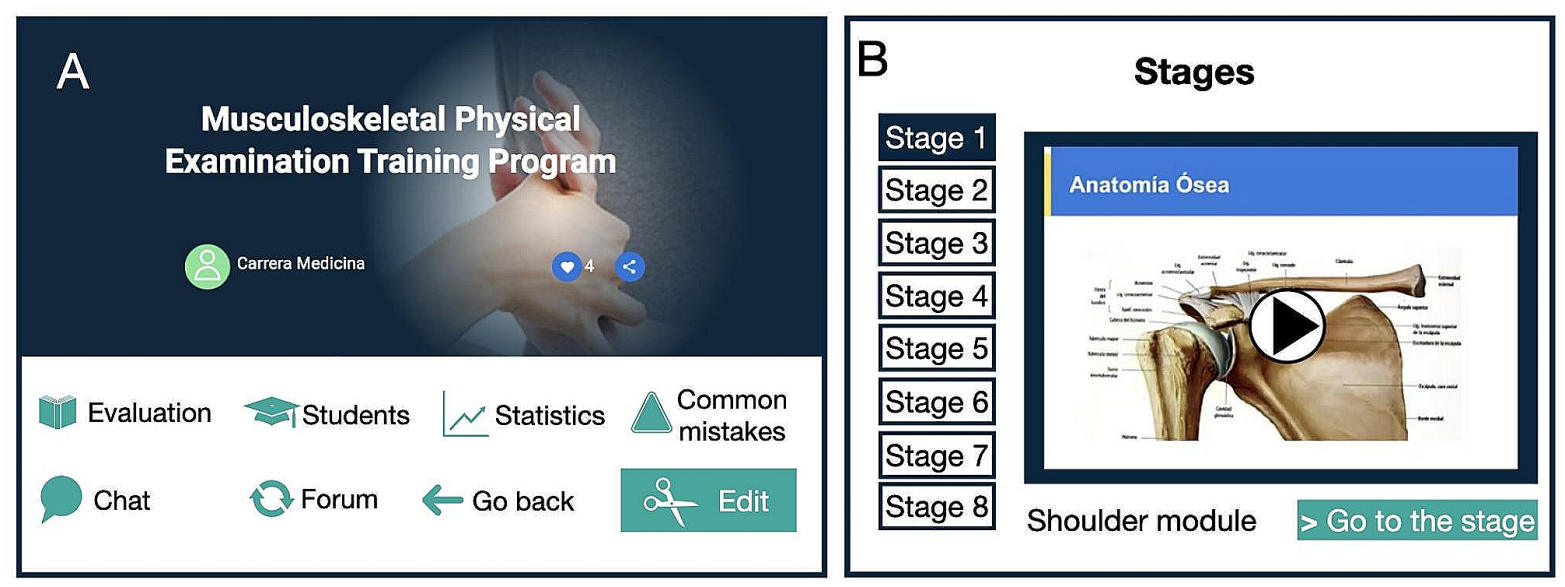
Musculoskeletal examination training module. **A**: Home Page, **B**: The prelude to the eight stages of the training module

**Fig. 2:**
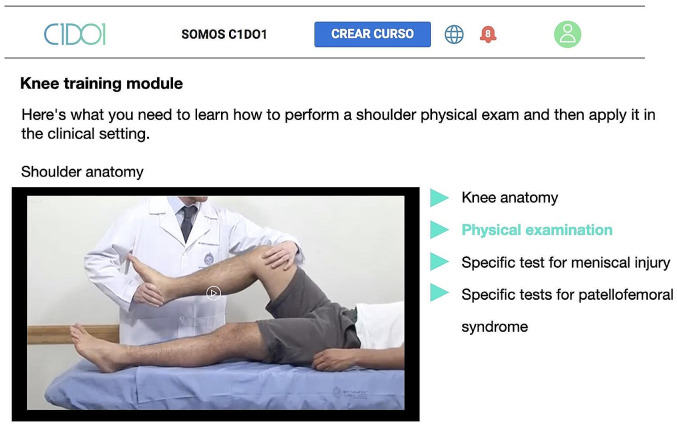
C1Do1 content. Lecture videos on anatomy, medical history, physical examination, and specific tests for common knee conditions

Using an experiential learning platform (C1Do1^®^, 15), a musculoskeletal examination training module was created (Fig. 1). The course included written and audiovisual content pertaining to medical history, physical examination, and specific tests for the diagnosis of the most common knee, spine, shoulder, ankle, and foot conditions (Fig. 2). The module was created and reviewed by a panel of experts consisting of eight orthopedic surgeons, including two surgeons per subspecialty. Priority pathologies, clinical cases (low back pain, patellofemoral pain, meniscal tear, ankle sprain, plantar fasciitis, rotator cuff injury), and evaluation guidelines were jointly agreed upon and reviewed twice by each specialist. Each module covers the anatomy, physiology, clinical presentation, physical examination, and specific tests for each joint through 5 videos of around 5–6 min each.

**Fig. 3:**
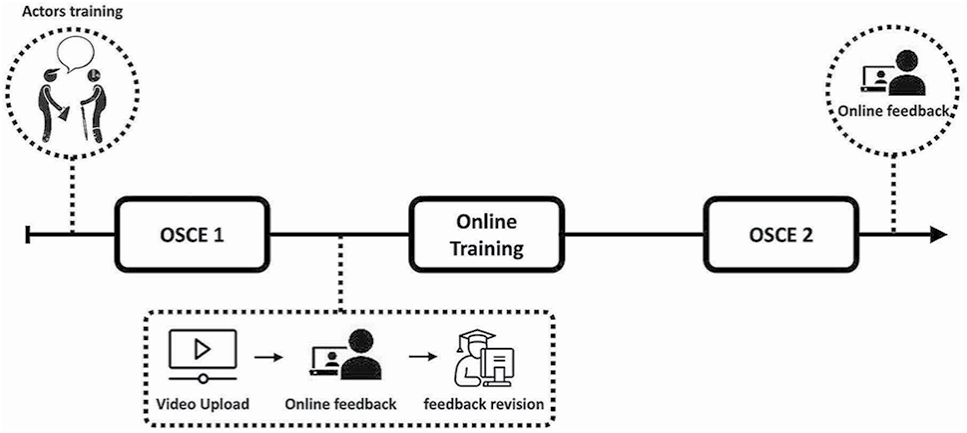
Musculoskeletal examination course methodology. Online training and feedback using an experiential learning platform (C1Do1^®^)

The four-week training module assessed interns’ physical examination skills using the objective structured clinical examination (OSCE) with simulated patients before and after completion took place face-to-face. Six clinical cases representing common musculoskeletal pathologies in primary care were created: SET A (Lumbar pain, meniscal tear, plantar fasciitis) and SET B (Ankle sprain, rotator cuff syndrome, patellofemoral pain). Participants randomly performed cases from SET A or SET B in the first OSCE and completed the remaining cases in the second OSCE. Three professional actors each simulated two clinical cases for a total of six cases, representing patients with these pathologies. All students encountered the same cases, differing only in their order of presentation across the two OSCEs, and examined the same three actors (Fig. 3).

**Fig. 4:**
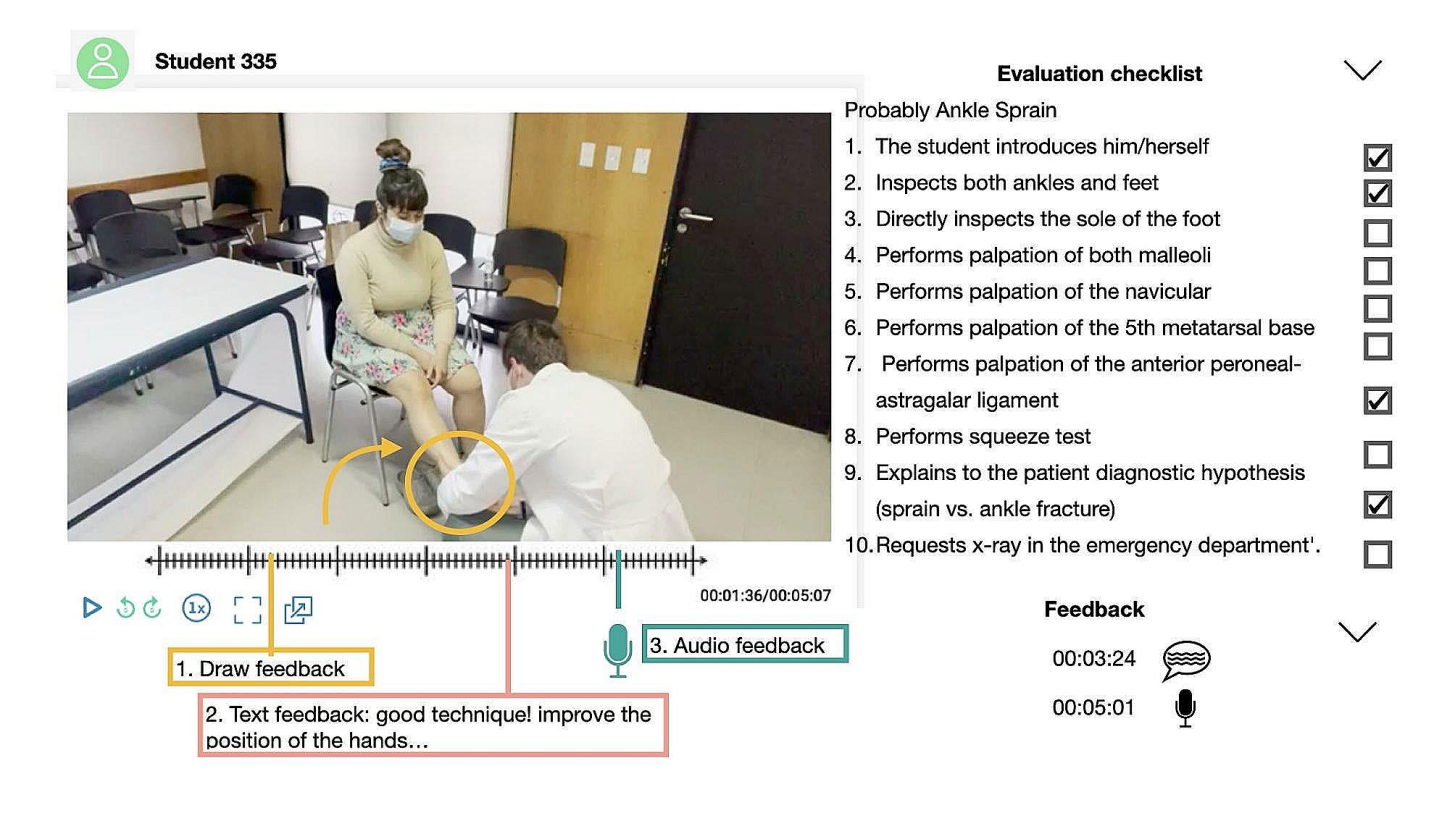
C1Do1 online platform. Teachers provide feedback of the student’s OSCE physical examination performance through audio, written commentary and/or drawings on the video image. Evaluation checklists were available to teachers for grading the student’s performance

The OSCEs were recorded, and the students were required to submit their videos via the online platform (C1Do1^®^). Six teachers, who were also orthopedic specialists, with at least 5 years of experience as an orthopedic surgeon, provided feedback during the following two weeks through written comments, audio recordings, and drawings (Fig. 4). They also completed a checklist created for each pathology. These checklists consisted of milestones that must be met for each musculoskeletal condition; possible scores ranged from 0 to 100%.

A control group of orthopedic specialists and senior residents were assessed using the OSCE, and their performance was compared with that of the interns before and after the training. At the conclusion of the study module and evaluations via the OSCEs, participants were queried as to their belief regarding the utility of the training module in their clinical practice and whether they would recommend the training module to a colleague.

Categorical variables are summarized in terms of absolute and relative frequencies, while numerical variables are shown as means and standard deviations. The normality of the data was determined using the Shapiro‒Wilk test. In the inferential analysis, paired T tests were performed to compare the means obtained by the students in the OSCE before and after completing the training module. T tests and Mann‒Whitney tests were conducted to compare the students’ baseline performance and the control group as well as the final performance with the control group. Stata software (v.16) was used to conduct statistical analysis, and *p* < 0.05 was considered to indicate statistical significance.

## Results

A total of 35 subjects participated in the first OSCE, including 29 interns and 6 experts. About 62% [15] of the interns were male and the median age was 24 (23–34). Of the experts, all were male, and the median age was 35 (32–58). At the end of the training module, 18 interns completed the second OSCE and 11 interns dropped out of the study. Teachers reviewed a total of 135 videos, including 85 videos from the first OSCE and 50 videos from the second OSCE. Four interns’ videos were excluded due to poor video quality.

The group of interns obtained a mean score of 50.6 ± 15.1 on the OSCE administered prior to the start of the module. This score increased significantly after the training to an average of 76.6 ± 12.8, *p* < 0.01.

**Fig. 5:**
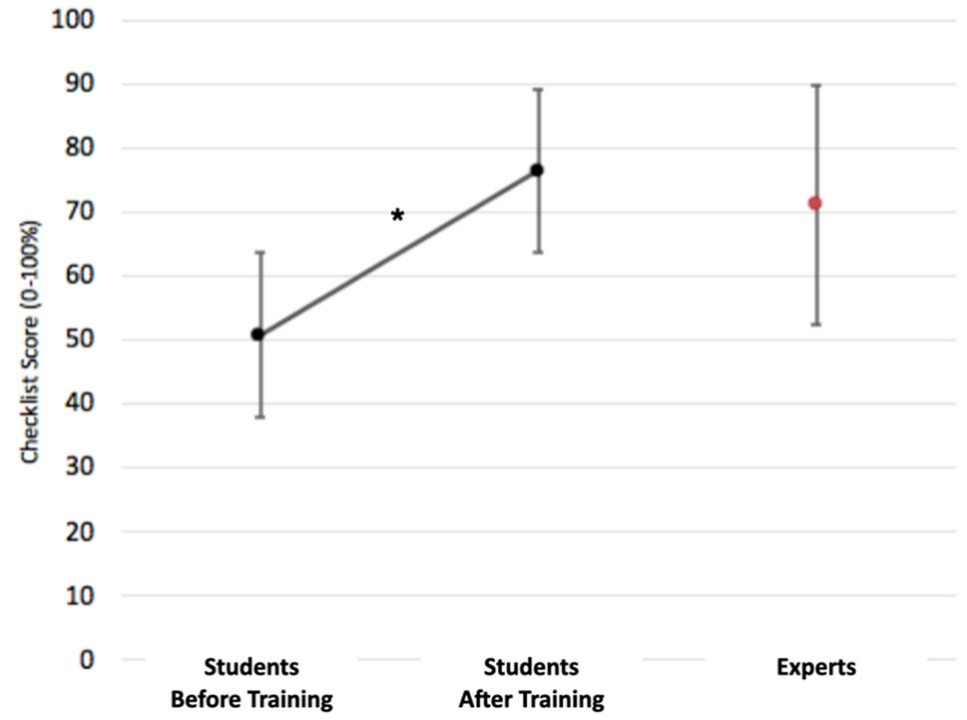
Students and expert performance before and after the online training module. *Statistically significant differences

Untrained interns performed significantly worse than the expert group (71.2 vs. 50.6; (*p* < 0.01). After receiving training and feedback, no significant differences were found between experts and the trained interns (71.2 vs. 76.6; *p* = 0.43) (Fig. 5).

Two evaluation questions were conducted at the end of the study, revealing that 93% of the participants affirm that the training module will be useful in their clinical practice, and 100% of the participants would recommend the training module to a colleague.

## Discussion

This study aimed to assess medical interns’ acquisition of musculoskeletal examination skills through their use of an online training and feedback tool. The results indicate that the online training and feedback module can enhance the musculoskeletal examination performance of medical interns. Clinical experience and scientific evidence have emphasized the importance of physical examination in the diagnosis of musculoskeletal disorders (4,). However, it has been reported that the training of general practitioners is insufficient [7, 8]. Our online training and feedback module enhances the musculoskeletal examination performance of medical interns and allows them to achieve significant improvements in their OSCE scores.

Several educational techniques for teaching musculoskeletal examinations have been developed. E-learning, a methodology that has gained prominence in recent years, has been shown to lead to similar or even superior results that those associated with traditional teaching methods [16]. In this study, the e-learning methodology was used to teach interns the necessary knowledge of the main musculoskeletal disorders over a short period of time.

Other studies have focused on different online platforms that make it possible to share audiovisual material, such as videos or podcasts, as well as to provide feedback on videos uploaded by students. In the study published in 2012 by Dr. Varas and his group, it was published how a laparoscopy training program and asynchronous feedback significantly improved laparoscopic skills in the simulation laboratory. Additionally, in their study published in 2017, it was demonstrated how these acquired skills were transferred to the operating room. There were 25 first-year residents who received both virtual and in-person feedback after training sessions or evaluations. [15, 17]. To foster the development of effective skills, this study integrated both e-learning and online feedback tools.On our platform, students can review the audiovisual material as many times as they need and pause video playback to memorize or take notes on fundamental concepts. Additionally, after each OSCE, they can review the feedback given by the teachers multiple times regarding their performance in each of the clinical scenarios.

The face-to-face OSCE sessions facilitated the assessment of physical examination performance as well as the inclusion of nontechnical aspects. These aspects play an important role in the learning process associated with the development of doctor‒patient communication in clinical practice [18]. It has been proven that nontechnical skills are trainable [19] and can benefit the acquisition of technical skills [20], so including the former in the learning context is nearly mandatory. The incorporation of a practice session with trained actors makes it possible to conduct a face-to-face evaluation of clinical skills in a protected environment as well as in an academic context.

The OSCE showed that the differences between students and experts prior to students’ completion of the training module could be overcome by training. Students began with a low average performance score during the initial session, revealing their lack of practice and training in physical examination. Following the completion of the training module and a comprehensive analysis of feedback provided by experts regarding the physical examination conducted during the initial OSCE, interns exhibited a notable improvement in their scores during the subsequent OSCE. Regardless of the method of feedback, the literature consistently notes that students who receive feedback learn more than their peers who train without it and require less practice time to achieve similar performance [21].”

This novel methodology is one of the strengths of this study, which proved that the online training and feedback module improves student performance. This module could be a useful and time-efficient tool for teaching clinical skills pertaining to physical examination without compromising patient safety or requiring teacher-student synchronization.

However, it is important to note that the study outcomes were based solely on OSCE scores evaluated through actor simulations. Future studies should consider incorporating feedback from real patients and comparing the validity of real case diagnoses with expert evaluations to better reflect true clinical utility and outcomes.

The study had limitations including the absence of a control group of interns using traditional teaching methods, although their initial evaluation reflected their knowledge at that stage. Other limitations included participant numbers and dropout rates. Major reasons for dropout and non-completion of the second OSCE were academic evaluations, rotations at other healthcare centers, and emergency department shifts. While the participant number was sufficient for analysis, it was limited in a university context. Efforts were made to contact participants and reschedule, but this was unsuccessful due to scheduling conflicts.

## Conclusion

The online training and feedback module significantly enhances the musculoskeletal examination performance of medical interns, leading to marked improvements in their OSCE scores. This study could be used as a starting point for the application of online training and feedback in multiple areas of medicine, specifically in the context of physical orthopedic examinations.

### Electronic supplementary material

Below is the link to the electronic supplementary material.

Supplementary Material 1

## Data Availability

The datasets used and analyzed during the current study are available from the corresponding author on reasonable request.

## Abbreviations

OSCE: Objective structured clinical examination
