# Supplementary material for: An online training and feedback module enhances the musculoskeletal examination performance of medical interns
Source: BMC Med Educ. 2024 Aug 23;24:917. doi: 10.1186/s12909-024-05683-w (PMC11344423; doi:10.1186/s12909-024-05683-w)
Supplement: Supplementary file 1 — Supplementary Material 1 [file 12909_2024_5683_MOESM1_ESM.docx]

Supplementary 1: checklists used to evaluate participants in the OSCE´s performed

| **Ankle Sprain** | **checklist** |
| --- | --- |
| The student introduces him/herself |  |
| Inspects both ankles and feet |  |
| Directly inspects the sole of the foot |  |
| Performs palpation of both malleoli |  |
| Performs palpation of the navicular |  |
| Performs palpation of the 5th metatarsal base |  |
| Performs palpation of the anterior peroneal-astragalar ligament |  |
| Performs squeeze test |  |
| Explains to the patient diagnostic hypothesis (sprain vs. ankle fracture) |  |
| Requests x-ray in the emergency department'. |  |

| **Plantar fasciitis** | **checklist** |
| --- | --- |
| The student introduces him/herself |  |
| Inspects hindfoot axis with the patient standing upright |  |
| Inspects the sole of the foot |  |
| Performs palpation of the plantar fascia insertion. |  |
| Performs palpation of the posterior tibial tendon pathway |  |
| Performs palpation of metatarsal heads |  |
| Performs palpation of the insertion of the achilles tendon |  |
| Performs palpation of the peroneal tendon trajectory. |  |
| Tests passive or active ROM of tibiotalar, subtalar and lisfranc joints |  |
| Compares ROM of affected foot with the contralateral foot |  |
| Performs Silfverskiöld test |  |
| Performs tinel test on the posterior tibial trajectory. |  |
| Explains the diagnostic hypothesis to the patient |  |

| **Meniscal Injury** | **checklist** |
| --- | --- |
| The student introduces him/herself |  |
| Identifies lower extremity alignment (standing or decubitus). |  |
| Performs patellar track |  |
| Performs wave or iceberg sign (any) |  |
| Performs passive ROM of both knees |  |
| Performs active ROM of both knees |  |
| Performs palpation of patella and anterior tibial tuberosity (must palpate both, otherwise consider NOT performed) |  |
| Perform palpation of both femoral condyles. |  |
| Performs palpation of both tibial plates. |  |
| Performs palpation of patellar tendon, quadricipital and goose foot (palpate all, otherwise consider NOT performed). |  |
| Performs palpation of both interlines |  |
| Performs brush or zohlen test. |  |
| Performs McMurray, Appley or Thessaly test. |  |
| Performs yawning in varus |  |
| Performs yawning in valgus |  |
| Performs lachman or anterior drawer test |  |
| Performs posterior drawer test |  |
| Compare any of the tests with the contralateral knee. |  |
| Explains the probable diagnosis to the patient |  |
| Explains to the patient the study or management plan associated with the probable diagnosis'. |  |

| **Patellofemoral Dysfunction** | **checklist** |
| --- | --- |
| The student introduces him/herself |  |
| Identifies lower extremity alignment (standing or decubitus). |  |
| Performs the wave or iceberg sign. |  |
| Performs passive ROM of both knees |  |
| Performs active ROM of both knees |  |
| Performs palpation of the patella and anterior tibial tuberosity (must palpate both, otherwise consider NOT performed). |  |
| Perform palpation of both femoral condyles. |  |
| Performs palpation of both tibial plates. |  |
| Performs palpation of patellar tendon, quadricipital and goose foot (palpate all, otherwise consider NOT performed). |  |
| Performs palpation of both interlines |  |
| Performs brush or zohlen test. |  |
| Perform McMurray, Appley or Thessaly test. |  |
| Compare any test with the contralateral knee. |  |
| Help the patient to sit up |  |
| Explains the probable diagnosis to the patient |  |
| Explains to the patient the study or management plan associated with the diagnosis'. |  |

| **Rotator Cuff Injury** | **checklist** |
| --- | --- |
| The student introduces him/herself |  |
| Inspects the symmetry of both shoulders |  |
| Performs palpation of the clavicle and acromioclavicular joint. |  |
| Performs palpation of pectoralis major, trapezius and cervical musculature |  |
| Performs palpation of the bicipital slide |  |
| Verify passive ROM (anterior elevation, anterior flexion, abduction, internal rotation, and external rotation). |  |
| Verifies active ROM (anterior elevation, anterior flexion, abduction, internal rotation and external rotation). |  |
| Performs Neer or Hawkins test |  |
| Performs Speed test, Yergason or Upper cut or similar. |  |
| Performs Jobe, Empty can or Full can or similar test |  |
| Performs external rotation test against resistance |  |
| Performs Bellypress Test, lift off or similar |  |
| Explains to the patient the diagnostic suspicion (rotator cuff injury) |  |
| Explains the study or management plan to the patient'. |  |

| **Low back pain** | **checklist** |
| --- | --- |
| The student introduces him/herself |  |
| Inspects sagittal and coronal balance |  |
| Palpates axial and paravertebral lumbar spine looking for tender points. |  |
| Asks the patient to perform hyperextension and hyperflexion of the spine. |  |
| Performs TEPE or lasegue test with the patient lying down. |  |
| Performs motor examination by roots from L2 to S1 |  |
| Performs sensory examination by roots from L2 to S1 |  |
| Examines patellar ROT |  |
| Examines Achilles ROT |  |
| Examines joint range of both hips |  |
| Tells the patient that what he probably has is lumbago |  |
| Performs education and indicates multimodal analgesia (both)'. |  |
